# Lithium chloride modulates macrophage polarization and enhances bone defect healing in the induced membrane technique by activation of Wnt/β-catenin signaling

**DOI:** 10.3389/fphar.2025.1621639

**Published:** 2025-10-21

**Authors:** Yong Qi, Wei Zhang, Ran Zhang, Shuanji Ou, Changpeng Xu, Yang Yang

**Affiliations:** Department of Orthopedics, The Affiliated Guangdong Second Provincial General Hospital of Jinan University, Guangzhou, China

**Keywords:** lithium chloride, induced membrane technique, macrophage polarization, Wnt/β-catenin signaling pathway, bone defect repair, osteogenic differentiation

## Abstract

**Background:**

The induced membrane technique (IMT) has become an effective method for treating bone defects; however, its efficacy is influenced by various factors. As an agonist of the Wnt/β-catenin cascade, lithium chloride (LiCl) has demonstrated notable osteogenic efficacy in recent years. Our current study systematically evaluated the therapeutic efficacy of LiCl in IMT-mediated bone reconstruction and the potential mechanisms by which it regulates macrophage polarization, osteoblast proliferation, and differentiation.

**Methods:**

Forty-eight male Sprague-Dawley rats were randomly assigned to 3 separate groups: high-dose LiCl (H-LiCl), low-dose LiCl (L-LiCl), and control group. A femoral bone defect model was established, and a PMMA spacer was subsequently implanted into the defect site in the first stage. After induced membrane formation, autologous bone grafts were implanted in the second stage. After 12 weeks, bone defect healing was evaluated by X-ray, micro-CT, and histological detection. Enzyme-linked immunosorbent assay (ELISA) and Immunohistochemical staining were used to evaluate the inflammatory cytokines in the induced membranes (IMs). Meanwhile, co-culture system consisting of RAW264.7 cells and bone marrow-derived mesenchymal stem cells (BMSCs) was used to assess the effects of LiCl on cell proliferation, macrophage polarization, osteogenic differentiation, and the Wnt/β-catenin pathway *in vitro*.

**Results:**

The high-dose LiCl group showed significant improvements in bone density, bone volume/total volume (BV/TV), and trabecular structure, exhibiting better bone repair outcomes. *In vitro*, LiCl markedly induced M2 macrophage polarization and enhanced BMSCs proliferation and mineralization.

**Conclusion:**

LiCl significantly improved bone regeneration and mineralization during bone defect repair by promoting M2 macrophage polarization, activating the Wnt/β-catenin pathway, and enhancing BMSCs differentiation.

## Introduction

Segmental bone defect reconstruction remains a formidable clinical predicament encountered in contemporary orthopedics, and particularly in cases involving severe trauma, infectious bone diseases, or tumor resection requiring volumetric tissue restoration (Alexander et al., 2011). In 1986, French investigators proposed a novel strategy involving the implantation of polymethylmethacrylate (PMMA) spacers at the defect site to stimulate development of a biologically active membrane structure (Barraza-Vergara et al., 2023; Bertacchini et al., 2018). This approach later evolved into the Induced Membrane Technique (IMT), which due to its high success rate in bone repair, has gradually become one of the mainstream methods for treating large bone defects (Collison, 2018; Frade et al., 2023; Fung et al., 2020). The induced membranes (IMs) not only provides a physical barrier that prevents premature degradation of the bone graft material, but also releases various growth factors and cytokines, including bone morphogenetic protein 2 (BMP-2), transforming growth factor-beta 1 (TGF-β1), and vascular endothelial growth factor (VEGF), which promote angiogenesis, osteoblast recruitment, and local inflammation regulation, thereby optimizing the bone regeneration process (Gessmann et al., 2022; Gindraux et al., 2017; Gindraux et al., 2020; Gohel et al., 2020).

Recent studies have shown that macrophages play a central role in formation of the membrane and bone repair. M1/M2 polarization not only influences the local inflammatory state but also regulates angiogenesis, osteogenic signaling, and bone matrix remodeling (Hachim et al., 2017; Han et al., 2017). Therefore, optimizing macrophage function and promoting macrophage polarization towards the osteogenesis-supportive M2 phenotype could be an important strategy for enhancing efficacy of the IMT. In bioinformatics analysis and differentially expressed genes (DEGs) and pathways in IMT, IMs was unique from all other tissues examined in that BMSCs were the most significantly enriched cell type and macrophages were the only immune cell type identified. The iPathwayGuide analysis indicated that the focus of the upregulated DEGs was on the signaling pathways related to those such as PI3K‐Akt and Wnt/β-catenin (Henrich et al., 2016). Macrophages are required for normal fracture healing and they impact BMSCs fate decisions and osteoblast differentiation in context-dependent ways (Kim et al., 2013).

Lithium chloride (LiCl), a widely used medication for treating bipolar disorder and epilepsy, has shown promise as an agent for use in bone regeneration and tissue engineering (Li et al., 2021). LiCl mainly functions by suppressing glycogen synthase kinase-3β(GSK3β) activity, which is a central brake in the Wnt/β-catenin signaling pathway that critically governs osteogenic differentiation and bone regeneration processes (Li et al., 2018; Li et al., 2005; Loi et al., 2016). Therefore, LiCl is generally considered to be an activator of the Wnt/β-catenin signaling pathway. Furthermore, LiCl has the immunomodulatory effects to alleviate inflammatory reactions and enhance the osteogenic differentiation of BMSCs by driving macrophage polarization (Marcol et al., 2020).

In fact, our understanding of immune modulation in PMMA-induced membranes is insufficient. Investigating the mechanisms underlying the effects of LiCl during different stages of the IMT, and particularly its roles in immune regulation, angiogenesis, and bone tissue remodeling, is crucial for optimizing bone defect repair strategies. This study investigated how LiCl modulates IM characteristics to enhance osseous defect repair. By integrating LiCl intervention with the IMT, a novel strategy was developed for enhancing the clinical efficacy of this technique, and thereby addressing critical needs in the management of complex bone defects.

## Materials and methods

### Establishment of animal models

The protocols for all animal studies were approved by the Ethics Committee of Guangdong Second Provincial General Hospital, and all animals were treated humanely according to national guidelines. A total of 48 Sprague-Dawley rats (male, 10–12 weeks old, mean body weight 300.5 ± 27.4 g) were randomly assigned to three groups: control (saline), low-dose LiCl (L-LiCl, 50 mg/kg/day), and high-dose LiCl (H-LiCl, 200 mg/kg/day), (n = 16 rats per group). LiCl (Sigma, St. Louis, Missouri, USA) was added to normal saline solution at a specific concentration. The dose of LiCl administered was determined by body surface area. Throughout the study period, biweekly weight measurements were used to guide real-time dose adjustments in response to physiological growth variations.

**FIGURE 1:**
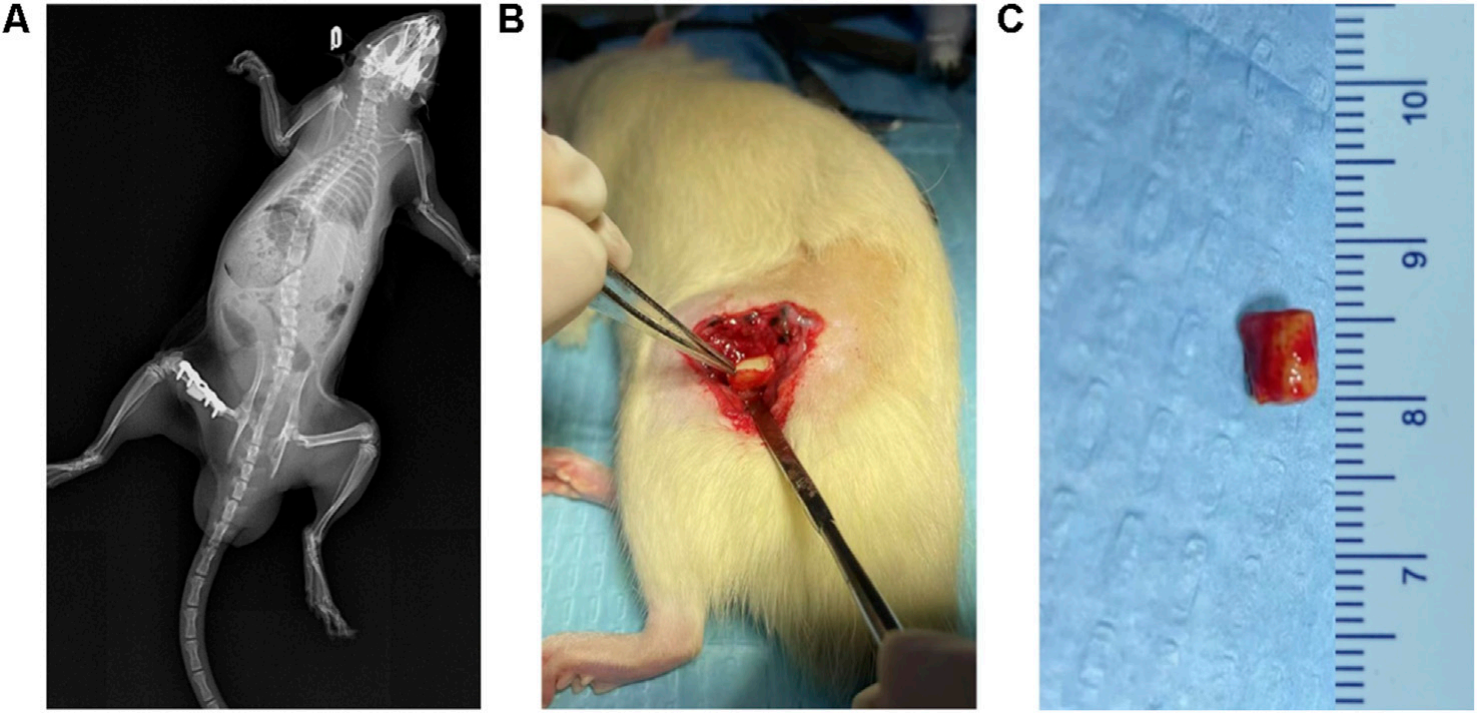
Establishment of IMT models. **(A)** X-ray images after the first stage of the IMT. The plate was firmly fixed and the PMMA Spacer was not displaced. **(B,C)** The inducted membrane on the surface of the PMMA Spacer.

The IMT was performed according to standard procedures similar to those used in the Masquelet rat protocol (Mauffrey et al., 2015). Prior to surgery, each animal received prophylactic penicillin (40,000 U) after a 12-h fasting period. After isoflurane anesthesia, the left hind limb was shaved for skin preparation. During the step I surgery, a longitudinal incision was made along the femoral axis, originating at the lateral greater trochanter and terminating at the lateral condyle of the femur, penetrating both the skin and fascial layers. This was followed by the separation of subcutaneous muscles, allowing the lateral femur to be exposed. A customized five-hole steel plate was placed on the lateral femur and fixed at its distal and proximal ends using 2 cortical self-tapping screws. Under continuous irrigation, a high-speed burr was used for osteotomy, creating a 5-mm segmental bone defect that was filled using a PMMA spacer (Figure 1A). Following step I surgery, LiCl or saline was orally administered each day for 4 weeks. At 4 weeks after step I surgery, 8 rats from each group were sacrificed, and the IM was collected for ELISA and Immunohistochemistry analysis (Figures 1B,C). The remaining rats underwent the step II surgery, during which two coccygeal vertebrae were harvested from the midportion of each rat tail, and granulized for bone grafting. Next, longitudinal cuts were made to the skin, subcutaneous tissues, and the IM along the surgical incision previously made in step I of the procedure. Following PMMA spacer extraction, the defect was reconstructed using particulate bone grafts. Finally, layered closure incorporated the IM, fascial layer, and cutaneous tissue with sutures. At 8 weeks after step II surgery, the rats were euthanized for bone defect analysis.

### X-ray and micro-CT analysis

At 8 weeks after step II surgery, X-ray imaging was performed to assess defect healing. The right femur was then harvested for micro-computed tomography (micro-CT) scanning and 3D reconstruction. The region of interest was manually defined to cover the entire transplanted bone area for subsequent analysis. For quantitative measurement of key parameters: relative bone volume (bone volume/total volume, BV/TV) was calculated as the ratio of mineralized bone volume to the total volume within the defined region of interest; bone mineral density (BMD) was quantified using a calibration phantom of known density to standardize and ensure the accuracy of measurements within the region of interest; trabecular morphology parameters (number, thickness, and separation) were also analyzed following the same region of interest definition.

### Histological and immunohistochemical analysis

After decalcification, bone defect specimens were paraffin-embedded and sectioned at 5-μm thickness for histological evaluation using hematoxylin and eosin (H&E) and Masson’s trichrome staining. Immunohistochemistry for the expression of BMP-2, β-catenin, p-GSK3β CD86(IMs) and CD163(IMs), was performed using standard procedures for deparaffinization, antigen retrieval, and peroxidase blocking. Sections were incubated overnight at 4 °C with specific primary antibodies (1:100, Abcam, Cambridge, MA, USA), followed by incubation with a secondary antibody and DAB staining.

### Preparation of PMMA nanoparticles

PMMA particles (80–90 nm diameter; Polysciences, Warrington, PA, USA) were processed according to the following standard sterilization protocol: (1) validation of endotoxin-free status by the Limulus amebocyte lysate assay, (2) triple sterilization in 70% ethanol (10 min per cycle), (3) extended decontamination via overnight incubation in 70% ethanol with 50 rpm orbital agitation, and (4) three washes with sterile phosphate-buffered saline (PBS).

### Cell culture

RAW264.7 macrophages were purchased from the China Center for Type Culture Collection (Shanghai, China), and rat BMSCs (rBMSCs) were separated from femur marrow aspirates according to established protocols. Both cell types were maintained in complete growth medium (Dulbecco’s Modified Eagle Medium, DMEM + 10% fetal bovine serum, FBS + 1% penicillin-streptomycin) in a humidified incubator at 37 °C with 5% CO_2_. Following seeding, the cells were exposed to PMMA nanoparticles (0.1 mg/mL) or LiCl at graded concentrations (0, 1, 5 mM), with medium renewal every 48 h. Cellular morphology was routinely monitored using an optical microscope (Leica, Germany).

### Cell proliferation assay

Cell viability was evaluated via the CCK-8 assay. RAW264.7 cells were plated into 96-well plates at an optimized density of 6 × 10^4^ cells per well (200 μL total volume/well). rBMSCs were seeded at a density of 3 × 10^4^ cells/well. After 1 and 4 days of treatment with PMMA nanoparticles and LiCl, the cells were washed three times with PBS and treated with 10% CCK-8 reagent at 37 °C for 4 h. Optical density at a wavelength of 450 nm was measured using a microplate reader.

### Immunofluorescence staining

Macrophage polarization status was evaluated by an immunofluorescence analysis of phenotypic markers (CCR7, C-C chemokine receptor type 7, for pro-inflammatory M1 polarization; Arg-1, arginase-1, for anti-inflammatory M2 polarization). Following 96-hours of culture, RAW264.7 cells were fixed with 4% paraformaldehyde and then blocked with 1% bovine serum albumin (BSA; 0.5 h) (30 min, room temperature). Next, the cells were incubated overnight at 4 °C with primary antibodies against CCR7 (1:100, Abcam) and Arg-1 (1:100, Abcam). The following day, the cells were washed with PBS and incubated with Alexa Fluor 594-conjugated donkey anti-mouse (1:200, Abcam) and Alexa Fluor 488-conjugated donkey anti-rabbit (1:200, Abcam) secondary antibodies in the dark for 1 h. The cell nuclei were stained with DAPI for 10 min, followed by imaging.

### Flow cytometry

To quantitatively analyze macrophage polarization, flow cytometry was performed to measure the expression of surface markers (CCR7 forM1 and Arg-1 for M2). After 4 days of culture, cells were collected, washed with PBS, and distributed in 1% BSA. The cells were then incubated with CCR7-APC (eBioscience, San Diego, CA, USA) and Arg-1-PE (eBioscience) antibodies for 1 h, while other groups of cells were incubated with corresponding isotype controls. Samples were acquired with a Guava flow cytometer (Millipore, Burlington, MA, USA) and analyzed using Guava software.

### Enzyme-linked immunosorbent assay (ELISA)

For *in vitro* cytokine analysis, the culture supernatants of RAW264.7 cells were harvested and clarified by centrifugation after 4 days of treatment. The concentrations of pro-inflammatory cytokines (TNF-α, IL-6) and anti-inflammatory mediators (IL-4, IL-10) were analyzed using commercially available ELISA kits (Anogen, ON, Canada) according to the manufacturer’s instructions.

In animal experiments, IM samples were collected at 4 weeks post-surgery, and ELISAs were performed to quantify the expression of those cytokines.

### Real-time quantitative PCR (RT-qPCR)

The total RNA from RAW264.7 cells was isolated using TRIzol. After quality assessment of the RNA, complementary DNA (cDNA) was synthesized using a RevertAid First Strand cDNA Synthesis Kit (Thermo Fisher, USA, Waltham, MA, USA). qPCR amplification was performed by using FastStart Universal SYBR Green Master Mix (Rox, Roche) on an ABI thermal cycler. The relative levels of CD86 and CD163 mRNA expression were calculated by the comparative Ct method and using *glyceraldehyde-3-phosphate dehydrogenase* (*GAPDH*) as a reference gene. The primer sequences used for RT-qPCR are listed in Table 1.

**TABLE 1:** Primer Sequences for Target Genes.

Gene		Primer sequence
CD86	F	AAC​TTA​CGG​AAG​CAC​CCA​CG
	R	CTT​TGT​AAA​TGG​GCA​CGG​CA
CD163	F	TGC​CTC​TGC​TGT​CAC​TAA​CG
	R	TTC​ATT​CAT​GCT​CCA​GCC​GT
GAPDH	F	GTG​TTC​CTA​CCC​CCA​ATG​TGT
	R	ATT​GTC​ATA​CCA​GGA​AAT​GAG​CTT

### Western blot analysis

The levels of protein expression in both RAW264.7 macrophages and rBMSCs after treatment with PMMA particles and/or LiCl were evaluated by Western blotting. Cells were lysed, and their total protein was harvested using radioimmunoprecipitation assay buffer (RIPA) (Yeasen, Shanghai) supplemented with a protease/phosphatase inhibitor cocktail. The lysates were maintained on ice for 30 min with periodic vortexing, and then clarified by centrifugation. The protein content of each lysate was measured using a BCA protein assay kit (Beyotime, China). Next, a 25 μg sample of protein from each lysate was separated by sodium dodecyl sulfate–polyacrylamide gel electrophoresis (SDS-PAGE), and the protein bands were subsequently electrotransferred onto Polyvinylidene fluoride (PVDF) membranes (Millipore).

The membranes were then blocked with 5% non-fat milk at room temperature for 1 h, and subsequently incubated overnight (4 °C) with primary antibodies specific for each cell type. For RAW264.7 cells, the detected proteins included BMP-2 and VEGF; whereas for rBMSCs, β-catenin, Cyclin D, c-Myc, osteopontin (OPN), osteocalcin (OCN), alkaline phosphatase (ALP), and Runx-2 were assessed. GAPDH served as an internal loading control in all experiments. Next, the membranes were incubated with a secondary antibody, and the immunoreactive bands were detected by exposure to a chemiluminescent substrate. The staining intensity of each protein band was quantified using ImageJ software.

### ALP and alizarin red staining (ARS)

After 2 weeks of culture, rBMSCs were fixed and stained with ALP and ARS (Beyotime). ALP activity was measured using an ALP assay kit (Beyotime) and normalized to the total protein content (BCA kit, Beyotime). ARS staining intensity was quantified by dissolving the dye in 10% cetylpyridinium chloride and measuring absorbance at 600 nm.

### Statistical analysis

All data are expressed as a mean value ± standard deviation (SD). All statistical analyses were performed using SPSS v17.0 software (IBM). Intergroup comparisons were performed using parametric tests (ANOVA for multiple groups, Student’s t-tests for pairwise comparisons). A P-value <0.05 was considered to be statistically significant.

## Results

### LiCl accelerated the growth rate of a bone graft

**FIGURE 2:**
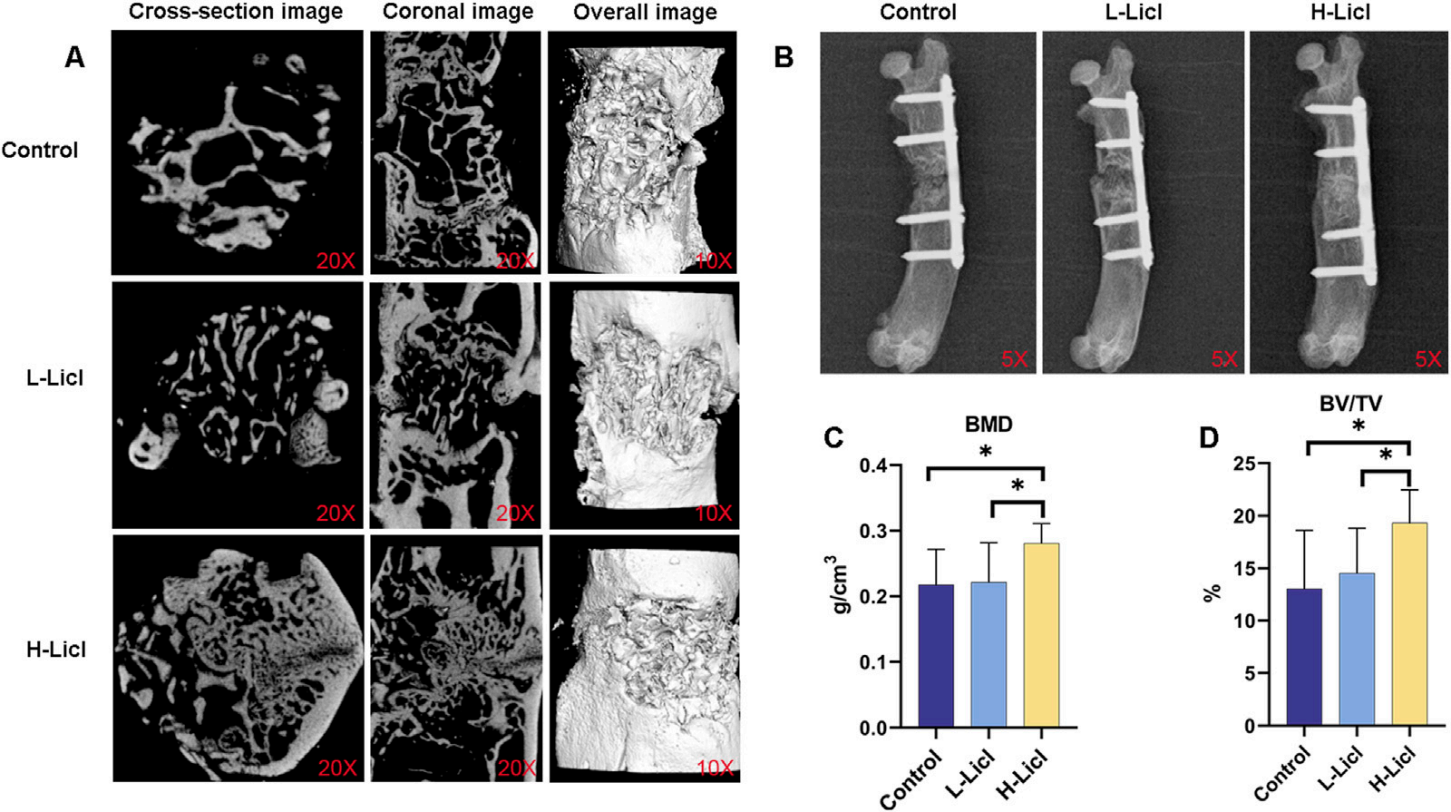
Micro-CT and X-ray analyses of bone graft and bone volume parameters. **(A)** Micro-CT images of bone defects in the control, L-LiCl, and H-LiCl groups, showing cross-sectional, coronal, and overall 3D reconstructed images of the bone healing area. **(B)** Representative X-ray images of bone healing in the control, L-LiCl, and H-LiCl groups after treatment. **(C,D)** Quantitative analysis of bone mineral density (BMD) and bone volume/total volume (BV/TV) ratio in the bone defect area (n = 3). *P < 0.05.

A Micro-CT analysis (Figure 2A) was performed to assess bone defect repair. The analysis showed markedly impaired osseous regeneration in control specimens, as evidenced by sparse and disorganized newly formed bone tissue, substantial void formation, and no clear evidence of bone bridging across the defect areas, indicating deficient bone repair capacity under baseline conditions. When compared to the control group, the L-LiCl group exhibited partial bone repair, with most of the defect area filled by trabecular bone, and mineralization was slightly improved. Meanwhile, the H-LiCl group showed almost complete closure of the defect area, with the formation of dense cortical bone. Moreover, the trabecular structure was uniform and well-organized, indicating that high-dose LiCl promoted better bone regeneration and remodeling.

X-ray imaging results (Figure 2B) showed the bone defect repair in rat femurs at 8 weeks after IMT surgery. The defect areas still had significant low-density images, with minimal periosteal callus formation and no obvious new bone bridging in the control group. The bone marrow cavity was partially closed but not fully continuous, suggesting limited bone regeneration. In the L-LiCl group, mixed-density images were observed in the defect area, with partial bone bridging and moderate mineralization at the fracture site, contributing to substantial restoration of the bone marrow cavity continuity. In contrast, the H-LiCl group exhibited clear high-density images, indicating more extensive mineralization and complete bone bridging. The fracture site was well-formed with prominent cortical bone formation, suggesting significantly enhanced bone regeneration and a more complete bone structure.

Quantitative analyses of BMD (Figure 2C) and BV/TV ratio (Figure 2D) further confirmed the stimulatory effect of LiCl on bone repair. The H-LiCl group showed significantly higher BMD and BV/TV values when compared with the L-LiCl and control groups, indicating substantial improvements in bone regeneration and mineralization. While the L-LiCl group also exhibited increased BMD and BV/TV values, the differences compared to the control group were not statistically significant, suggesting that the promoting effect of low-dose LiCl on bone repair was relatively weak.

### LiCl upregulated the Wnt/β-catenin signaling pathway and remodeled the bone graft

**FIGURE 3:**
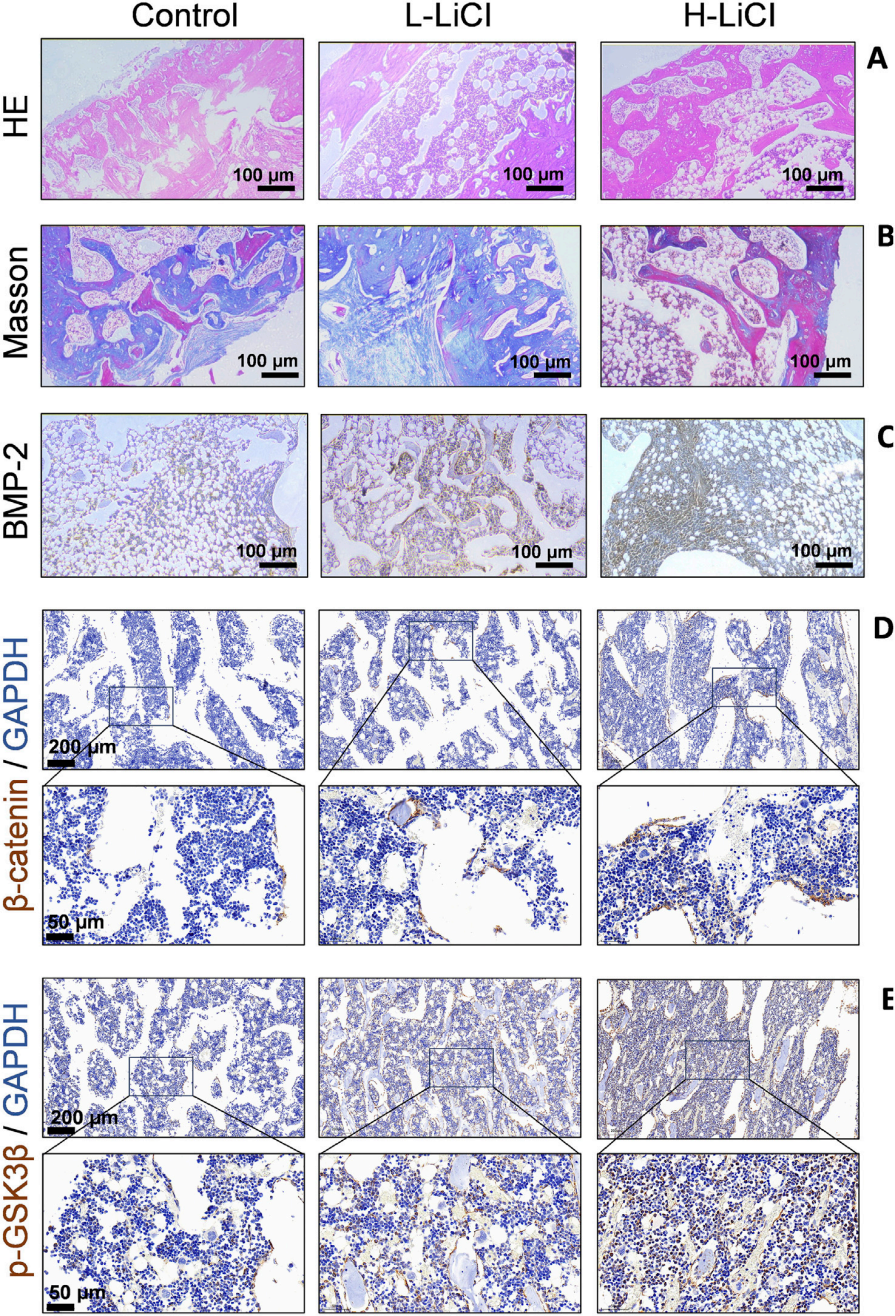
The effect of LiCl on bone graft. **(A)** Histological structure of the bone healing area in the control, L-LiCl, and H-LiCl groups as analyzed by hematoxylin and eosin (H&E) staining. **(B)** Collagen deposition and bone formation in the defect areas of the control, L-LiCl, and H-LiCl groups as analyzed by Masson’s trichrome staining. **(C–E)** Immunohistochemical staining for BMP-2,β-catenin and p-GSK3β in the bone defect areas of the control, L-LiCl, and H-LiCl groups.

Histological changes in the bone defect area were detected by H&E and Masson staining (magnification ×100; Figures 3A,B). The defects in the control group exhibited predominant fibrous tissue deposition, few newly formed chondrocytes, and sparse cortical bone with minimal remodeling. In the L-LiCl group, bone-like tissue was observed in the marrow cavity with some microvessel formation and inflammatory infiltration; however, cortical bone remodeling was incomplete. In contrast, the H-LiCl group showed significant bone regeneration, with organized trabecular bone, fewer fibrous tissues, and a thicker cortical bone, indicating a dose-dependent effect of LiCl in promoting bone repair.

Immunohistochemical staining (Figures 3C–E) was performed to detect BMP-2,β-catenin and p-GSK3β expression in the bone defect areas of the different treatment groups. In the control group, staining for BMP-2 expression was weak, indicating low osteogenic activity. The L-LiCl group exhibited moderate BMP-2 staining, suggesting some degree of bone regeneration. In contrast, the H-LiCl group showed strong and widespread staining for BMP-2 expression, indicating that LiCl promoted osteogenic activity and that high-dose LiCl significantly increased BMP-2-mediated bone regeneration (Figure 3C). LiCl treatment enhanced the expression of β-catenin and p-GSK3β in bone defect areas in a dose-dependent manner (Figures 3D,E), the inactivation (phosphorylation) of GSK3b leads to dephosphorylation of β-catenin and activation of Wnt signaling to promote bone anabolism.

### LiCl inhibited the expression of inflammatory cytokines in IMs

**FIGURE 4:**
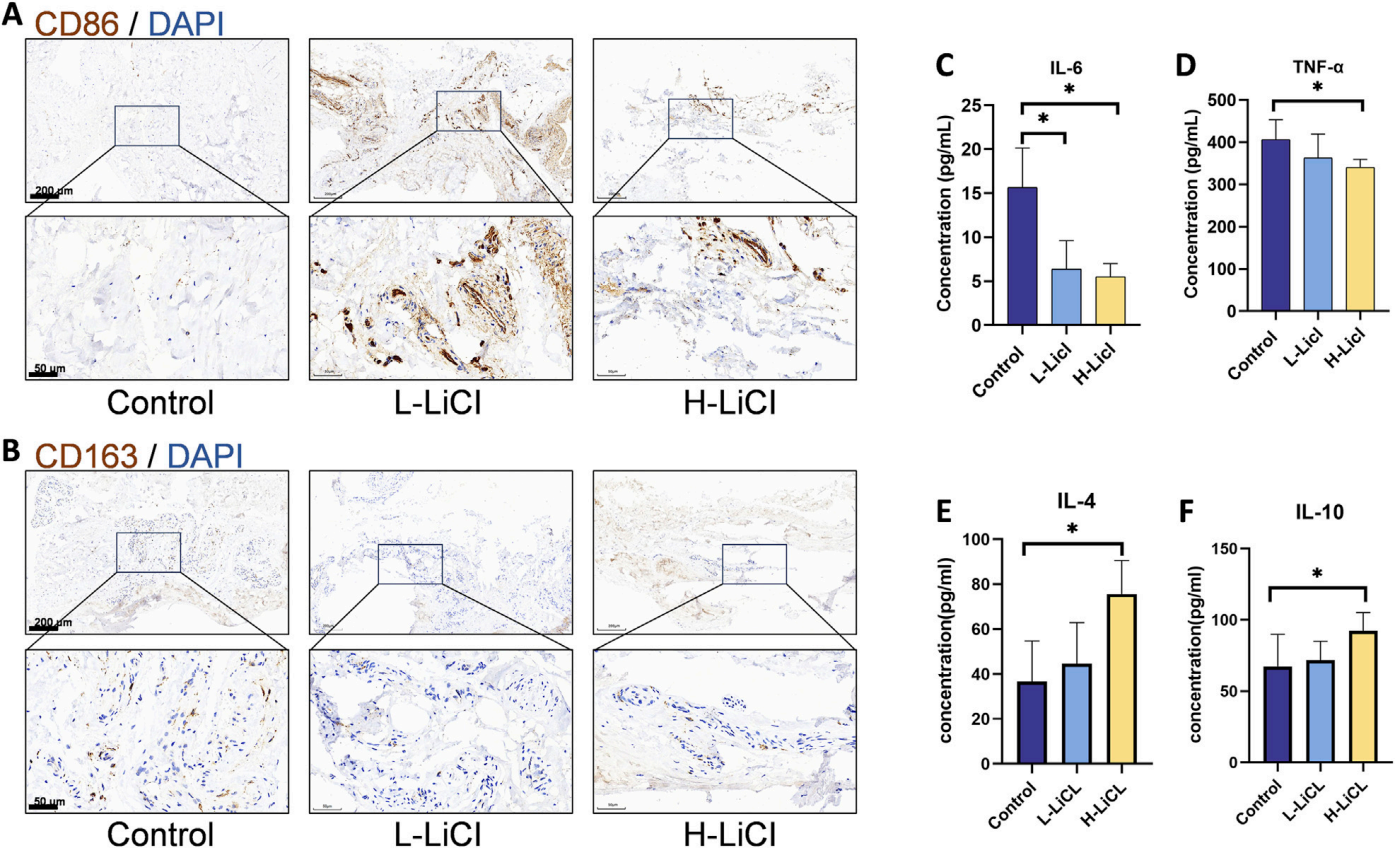
The effect of LiCl on induced membranes (IMs). **(A,B)** The expression of CD86 and CD163 in the IMs detected by immunohistochemical staining. **(C–F)** ELISA results showing IL-6, TNF-α, IL-4 and IL-10 concentrations in the IMs (n = 3). *P < 0.05.

Immunohistochemical staining for the IMs of the different treatment groups revealed that CD86(the M1 macrophage marker) expression was decreased and CD163(the M2 macrophage marker) was increased in H-LiCl group (Figures 4A,B). To further investigate the anti-inflammatory property of LiCl in IMs, the expression levels of IL-6, TNF-α, IL-4 and IL-10 in the IMs were measured by ELISA. As depicted in Figures 4C–F, IMs in the H-LiCl group released the highest amounts of IL-4 and IL-10(anti-inflammatory cytokines), which are mainly generated by M2 macrophages. However, the secretion of inflammatory cytokines, TNF-α and IL-6(generated by M1 macrophages), were the highest in the Control group.

### Cell morphology and proliferation analysis

**FIGURE 5:**
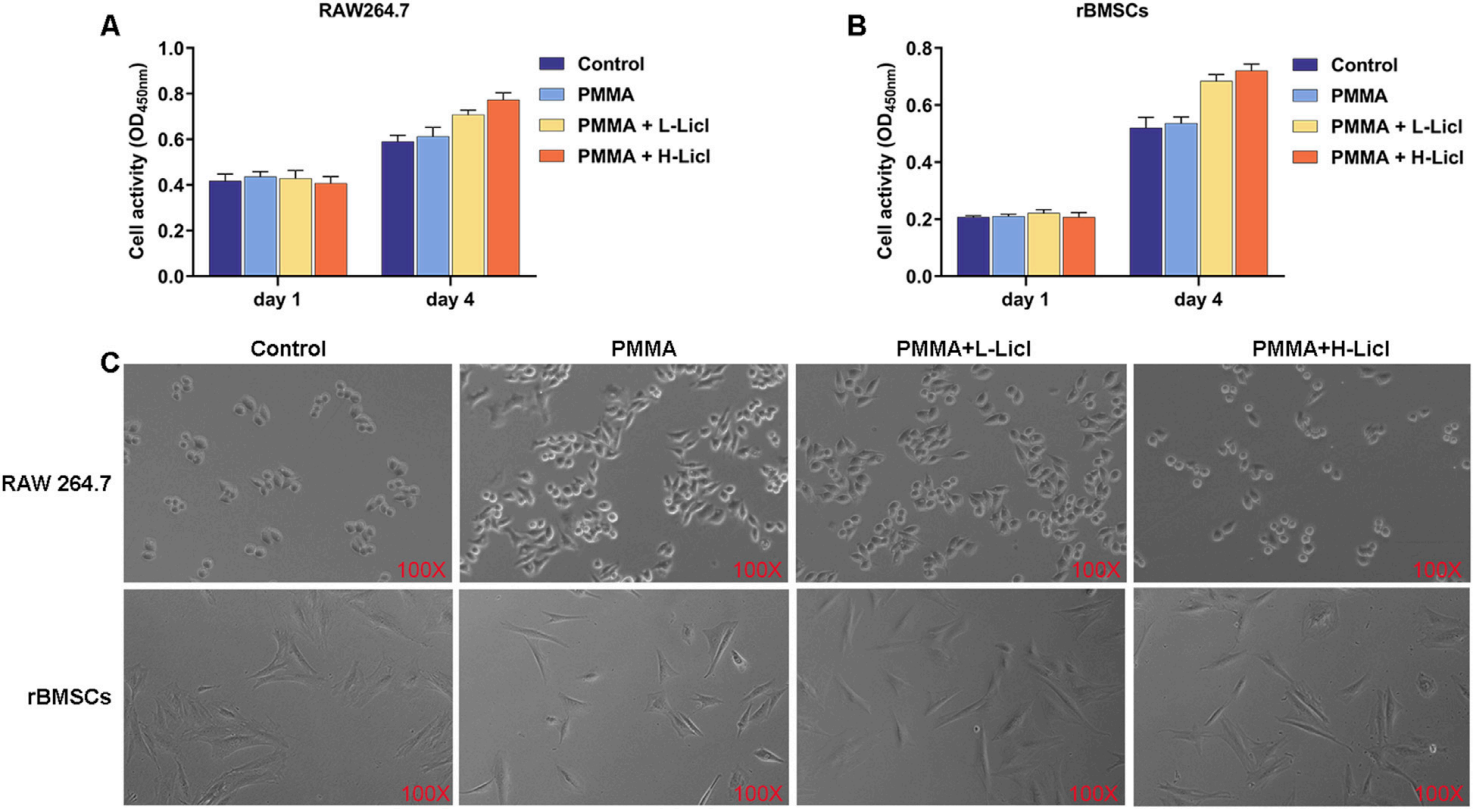
Cell proliferation analysis of RAW264.7 macrophages and rBMSCs after different treatments. **(A)** The cellular activity of RAW264.7 macrophages on days 1 and 4 as measured by the CCK-8 assay. **(B)** The activity of rBMSCs on days 1 and 4 as measured by the CCK-8 assay. **(C)** Representative phase contrast images of RAW264.7 macrophages and rBMSCs undergoing different treatments at day 4. The upper row shows RAW264.7 macrophages, and the lower row shows rBMSCs.

The effects of PMMA particles and LiCl on the proliferation of RAW264.7 macrophages and rBMSCs were evaluated by the CCK-8 assay, and cell morphology was observed using an optical microscope. The proliferation assays (Figures 5A,B) revealed no significant differences in cell activity among the groups at days 1 and 4, indicating that the PMMA and LiCl treatments were not notable toxic to the RAW264.7 cells and rBMSCs. Furthermore, optical microscopy (Figure 5C) showed that in the control group, the RAW264.7 cells were round in shape; however, after PMMA stimulation, the cells became flattened with increased protrusions. Following high-concentration LiCl treatment, the percentage of polygonal cells decreased, and the percentage of spindle-shaped cells increased. However, there were no significant morphological changes in rBMSCs after treatment with PMMA or LiCl, suggesting that those treatments had minimal impact on the cell shape of rBMSCs.

### LiCl drove macrophage phenotype switching from M1 to M2 polarization

**FIGURE 6:**
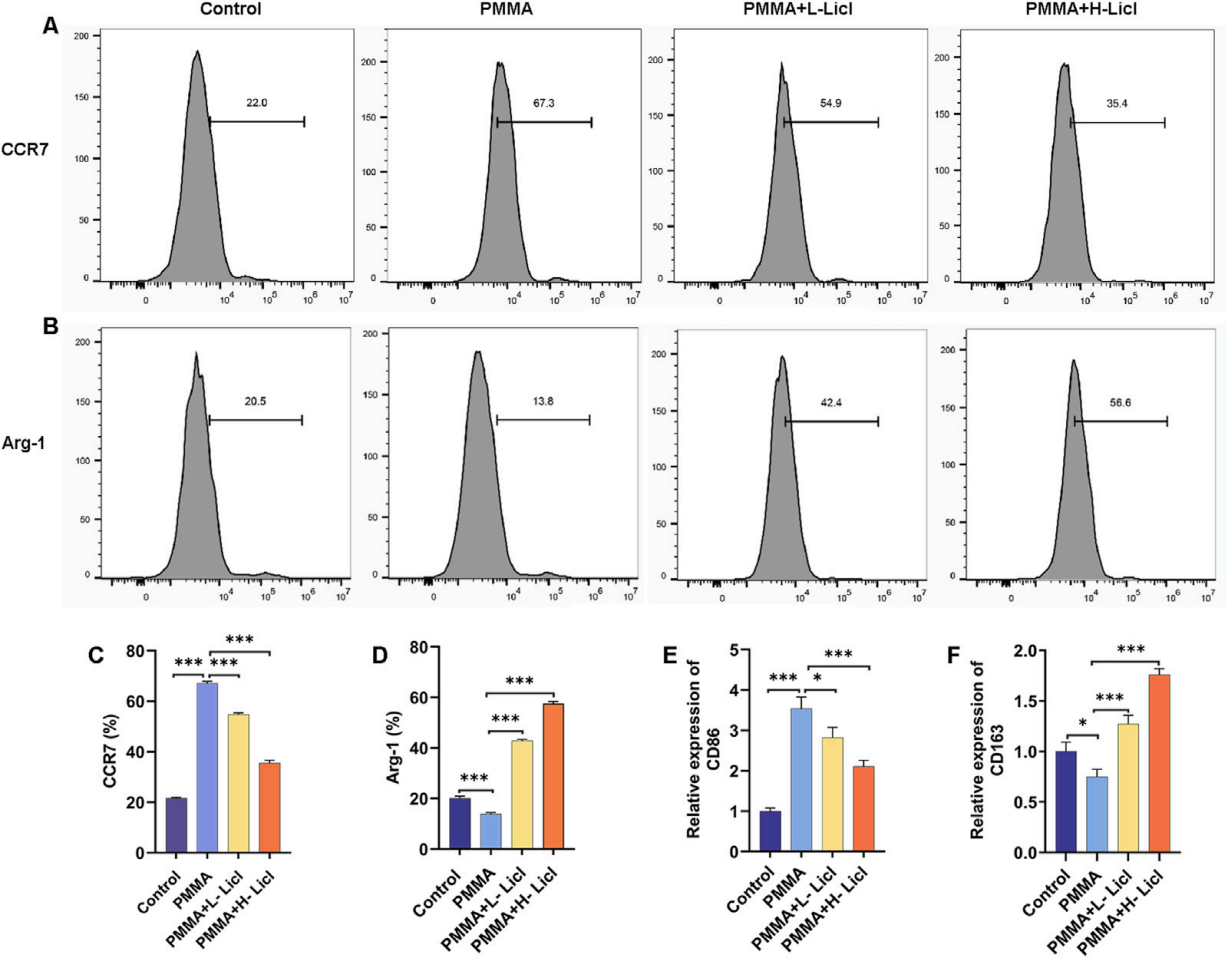
Flow cytometric and PCR analyses of macrophage polarization in response to PMMA particles and LiCl treatment. **(A,B)** Expression of the M1 macrophage marker CCR7 and the M2 macrophage marker Arg-1 in RAW264.7 cells was detected by flow cytometry. **(C,D)** Quantification of CCR7 and Arg-1 expression in RAW264.7 cells as a percentage of total cells. **(E,F)** Relative levels of CD86 (M1) mRNA expression and CD163 (M2) mRNA expression in RAW264.7 cells, as assessed by qPCR. *P < 0.05, **P < 0.01, *P < 0.001.

Flow cytometry analyses (Figures 6A–D) showed that CCR7 expression (M1) was significantly upregulated after PMMA treatment when compared with the control group. However, following LiCl treatment, CCR7 expression gradually decreased, with the lowest expression in the H-LiCl group. In contrast, Arg-1 (M2) expression was reduced in the PMMA group, whereas in the LiCl treatment groups, Arg-1 expression exhibited a dose-dependent increase, with the highest expression in the H-LiCl group. These results suggest that LiCl treatment promoted M2 polarization (upregulation of Arg-1) and inhibited M1 polarization (downregulation of CCR7) in dose-dependent manners.

To further clarify the effects of PMMA and LiCl on macrophage polarization, we assessed the levels of CD86 (M1) and CD163 (M2) expression by qPCR (Figures 2F, 6E). When compared with the control group, CD86 expression was significantly upregulated after PMMA treatment, and showed a dose-dependent decrease in the LiCl-treated groups, with its lowest expression observed in the H-LiCl group (Figure 6E). Conversely, CD163 expression was lowest in the control group, increased after PMMA treatment, and then increased further following LiCl treatment, with its highest expression observed in the H-LiCl group (Figure 6F). These results further confirmed that LiCl treatment promoted M2 macrophage polarization and inhibited the characteristic expression of M1 macrophages.

**FIGURE 7:**
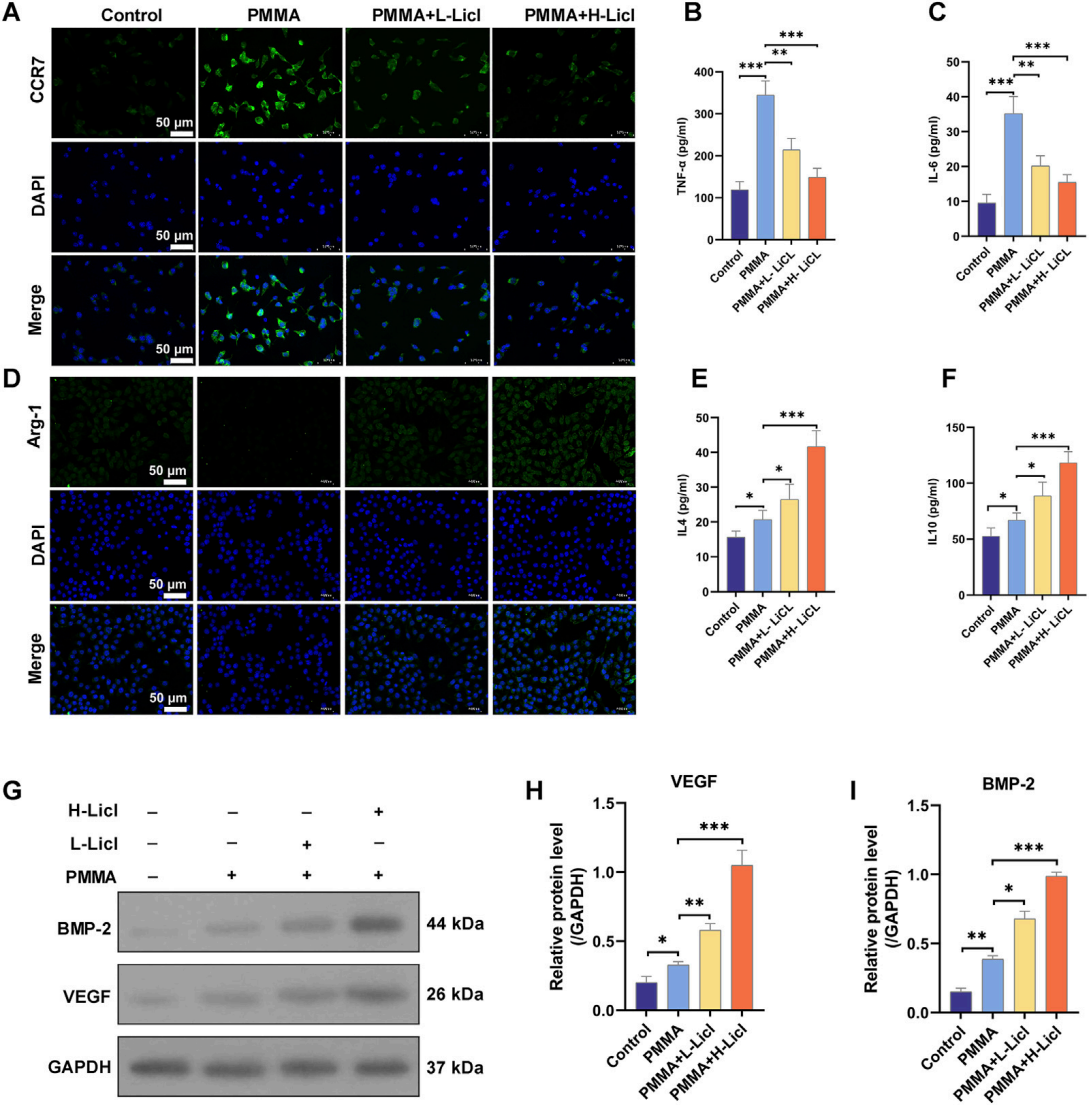
Immunofluorescence and cytokine analyses of macrophages. **(A)** Representative immunofluorescence images showing the expression of CCR7 in RAW264.7 cells. Nuclei are stained with DAPI (blue), and CCR7 is shown in green. **(B,C)** ELISA analysis of TNF-α and IL-6 levels in cell supernatants. **(D)** The expression of Arg-1 in RAW264.7 cells as analyzed by immunofluorescence. Nuclei are stained with DAPI (blue), and Arg-1 is shown in green. **(E,F)** ELISA analysis of IL-4 and IL-10 levels in cell supernatants. **(G)** Representative Western blot analysis of BMP-2, VEGF, and GAPDH expression in RAW264.7 cells. **(H,I)** Quantification of BMP-2 and VEGF protein expression relative to GAPDH. *P < 0.05, **P < 0.01, *P < 0.001.

To evaluate the effects of different treatments on macrophage polarization, we detected the expression of CCR7 (green) and Arg-1 (green) in RAW264.7 cells, with DAPI (blue) staining the cell nuclei (Figures 7A,D). The results showed that CCR7 expression was low in the control group, but significantly upregulated after PMMA treatment. However, in the LiCl treatment groups (especially in the PMMA + H-LiCl group), the numbers of CCR7-positive cells were markedly reduced, indicating that LiCl inhibited M1 polarization (Figure 7A). Conversely, Arg-1 expression was low in the control group and slightly decreased after PMMA treatment, but the number of Arg-1 positive cells was significantly increased in the PMMA + LiCl groups, and particularly in the high-concentration LiCl group (Figure 7D), indicating that LiCl promoted M2 polarization.

### LiCl promoted the secretion of anti-inflammatory cytokines and osteogenesis-related proteins by macrophages

To characterize the immunomodulatory effects of PMMA and LiCl, we quantified the cytokines secreted by cultured RAW264.7 macrophages (Figures 7B,C,E,F). Our results showed that TNF-α and IL-6 were expressed at low levels in the control group, but their expression was significantly upregulated after PMMA treatment, indicating that PMMA promotes inflammation. However, LiCl treatment dose-dependently attenuated the secretion of both TNF-α and IL-6 (Figures 7B,C), suggesting that LiCl alleviates PMMA-induced pro-inflammatory responses. In contrast, IL-4 and IL-10 production showed an opposite trend to that of the pro-inflammatory cytokines (Figures 7E,F). After PMMA treatment, IL-4 and IL-10 levels were slightly increased, whereas in the PMMA + LiCl groups (especially in the high-concentration LiCl group), both IL-4 and IL-10 expression were significantly upregulated, indicating that LiCl promotes anti-inflammatory responses.

BMP-2 and VEGF expression in RAW264.7 cells were detected by western botting, with GAPDH serving as an internal control. As shown in Figures 7G,H, BMP-2 expression was promoted by PMMA treatment, and further upregulated in the PMMA + L-LiCl group, with the highest levels of BMP-2 expression observed in the PMMA + H-LiCl group. Similarly, VEGF expression (Figure 7I) was significantly increased after PMMA treatment, and LiCl further enhanced VEGF expression, with its highest levels observed in the PMMA + H-LiCl group.

In conclusion, PMMA primarily induced pro-inflammatory responses, while LiCl modulated the inflammatory microenvironment by inhibiting pro-inflammatory cytokine production and enhancing anti-inflammatory cytokine secretion. Meanwhile, the increasing in BMP-2 and VEGF expression demonstrated the potential osteogenesis effects of LiCl.

### LiCl upregulated the Wnt/β-catenin signaling pathway and promoted BMSC maturation

**FIGURE 8:**
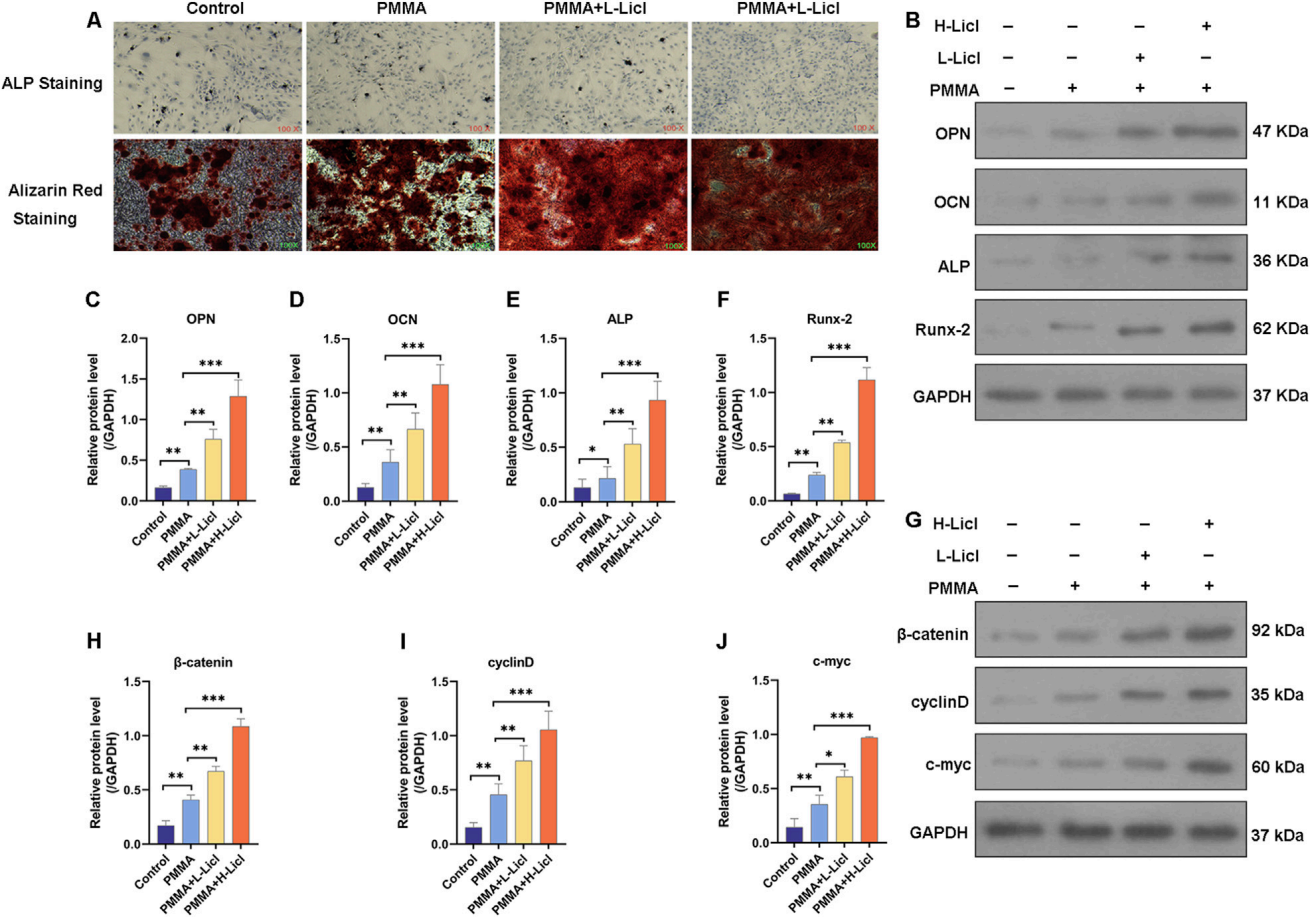
Assessment of osteogenic differentiation and Wnt/β-catenin pathway activation in BMSCs. **(A)** Representative images of alkaline phosphatase (ALP) staining (top) and Alizarin Red S staining (bottom) of BMSCs. **(B)** Western blot analysis of osteogenesis-related proteins OPN, OCN, ALP, and Runx-2. **(C–F)** Quantification of OPN, OCN, ALP, and Runx-2 protein levels relative to those of GAPDH. **(G)** Western blot analysis of β-catenin, cyclin D, and c-myc expression. **(H–J)** Quantification of β-catenin, cyclin D, and c-myc protein levels relative those of GAPDH. *P < 0.05, **P < 0.01, ***P < 0.001.

ALP and ARS staining studies were performed to assess the osteogenic capacity of BMSCs in the different treatment groups (Figure 8A). When compared with the control group, PMMA slightly enhanced mineral deposition and ALP activity. The addition of low-dose LiCl (PMMA + L-LiCl) further increased mineralization and ARS staining intensity, whereas the high-dose group (PMMA + H-LiCl) exhibited the most prominent red and purple staining, indicating a dose-dependent enhancement of osteogenic differentiation by LiCl.

Western blotting revealed that PMMA alone moderately increased the expression of OPN, OCN, ALP, and Runx-2 when compared with the control group. Moreover, LiCl co-treatment further upregulated those markers in a dose-dependent manner, with the PMMA + H-LiCl group showing the highest expression levels (Figures 8B–F).

Furthermore, the expression of key components in the Wnt/β-catenin pathway (β-catenin, cyclin D, and c-myc) was evaluated (Figures 8G–J). PMMA treatment increased the expression of those proteins compared to the control, and LiCl further enhanced their expression; particularly in the high-dose group. These results suggest that LiCl promotes osteogenesis partially by activating the Wnt/β-catenin signaling pathway.

### M2 macrophage polarization enhances osteogenic differentiation of BMSCs

**FIGURE 9:**
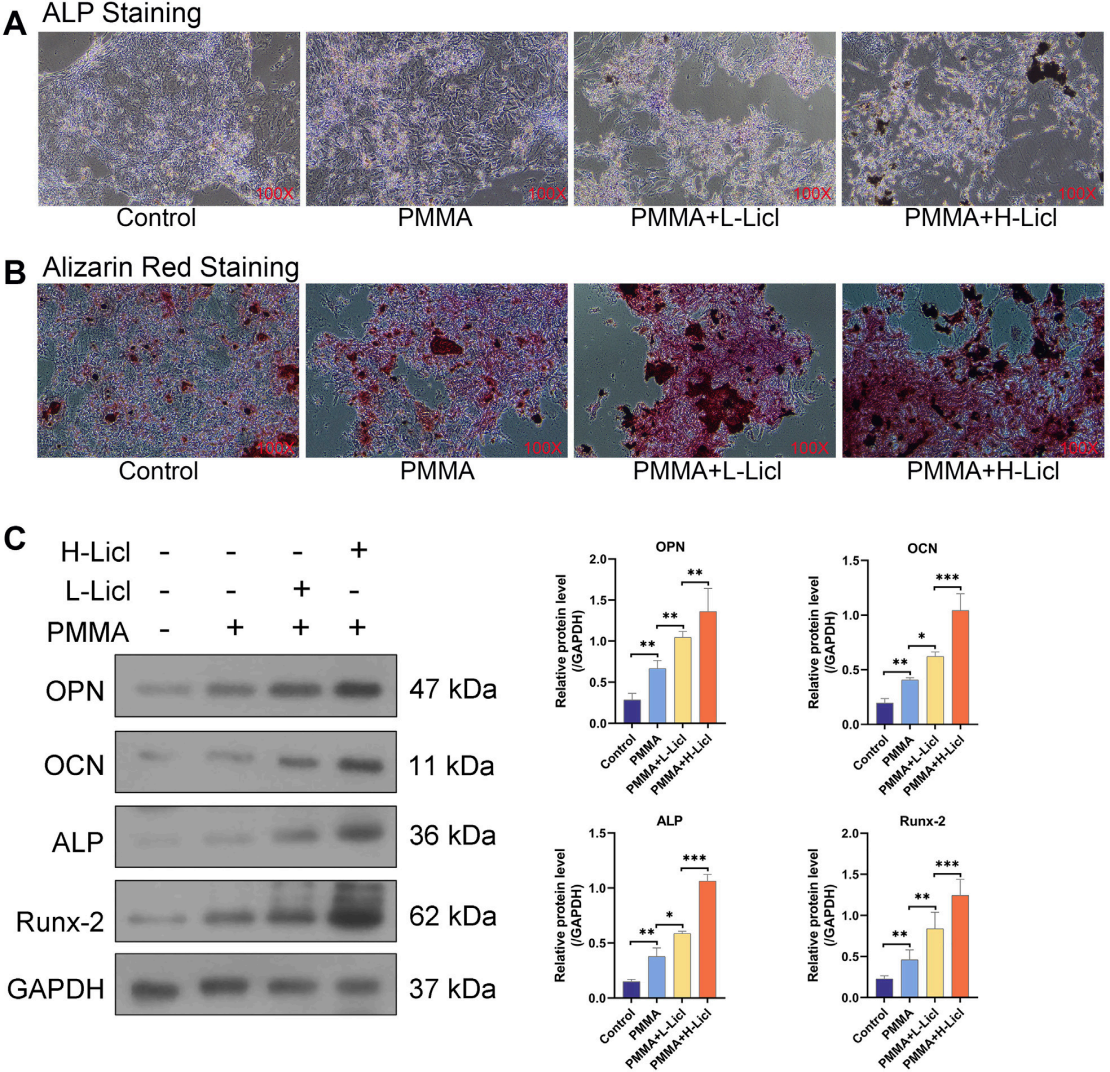
The effect of macrophage polarization on BMSCs differentiation. **(A)** ALP staining of BMSCs. **(B)** ARS staining of BMSCs. **(C)** Western blot analysis of osteogenesis-related proteins OPN, OCN, ALP, and Runx-2. *P < 0.05, **P < 0.01, ***P < 0.001.

To investigate the influence of macrophage polarization on the osteogenic differentiation of BMSCs, we co-cultured BMSCs with differentially treated RAW264.7. Osteogenic capability was evaluated using ALP and ARS staining. Compared to the control group, RAW264.7 treated with PMMA alone slightly enhanced mineral deposition and ALP activity in BMSCs (Figure 9A). RAW264.7 treated with both PMMA and L-LiCl further promoted matrix mineralization and intensified ARS staining (Figure 9B). The most pronounced effects were observed in the PMMA plus H-LiCl group, which exhibited the strongest red and purple staining. Furthermore, Western blot analysis revealed a dose-dependent upregulation of the expression of OPN, OCN, ALP, and Runx2 in BMSCs across the treatment groups (Figure 9C), consistent with the staining results. These results suggested that M2 macrophages enhanced the osteogenic differentiation of BMSCs.

## Discussion

The IM differed from both the granulation tissue at a bone defect site and the membrane formed by a pure foreign body response. It contained large amounts of osteogenic and angiogenic factors, including bone morphogenetic protein-2 (BMP-2) and vascular endothelial growth factor (VEGF). The foreign body response to polymethylmethacrylate (PMMA) at the bone defect site was a necessary condition for IM formation. Crosstalk between macrophages and rBMSCs might explain the mechanism of IM formation (Minardi et al., 2016). Emerging evidence highlights macrophages as pivotal regulators of fracture repair, and suggests their immunomodulatory crosstalk with BMSCs is involved in immune regulation (Morwood et al., 2019). Despite having an incomplete understanding of the mechanism (Muneer, 2017; Nauta and Fibbe, 2007), this crosstalk has emerged as a promising therapeutic target for enhancing bone regeneration.

Strategies proposed to accelerate bone healing include enhancing macrophage recruitment to the fracture site (Pajarinen et al., 2019; Raggatt et al., 2014) and modulating the phenotypic polarization of macrophages toward the M2 subset (Roddy et al., 2018; Schlundt et al., 2021; Song et al., 2023). Research has shown that amplification of macrophage-mediated inflammatory signaling during the early phase of a fracture can increase BMSC recruitment to an injury site, and thereby enhance the osteogenic potential of BMSC-derived bone regeneration (Tang et al., 2014). Optimal bone repair requires a precisely orchestrated immunoregulatory sequence of events beginning with M1 macrophage-driven transient inflammation, followed by progressive M2 polarization. This sequence of events creates an ideal immune microenvironment for bone regeneration (Schlundt et al., 2021).

**FIGURE 10:**
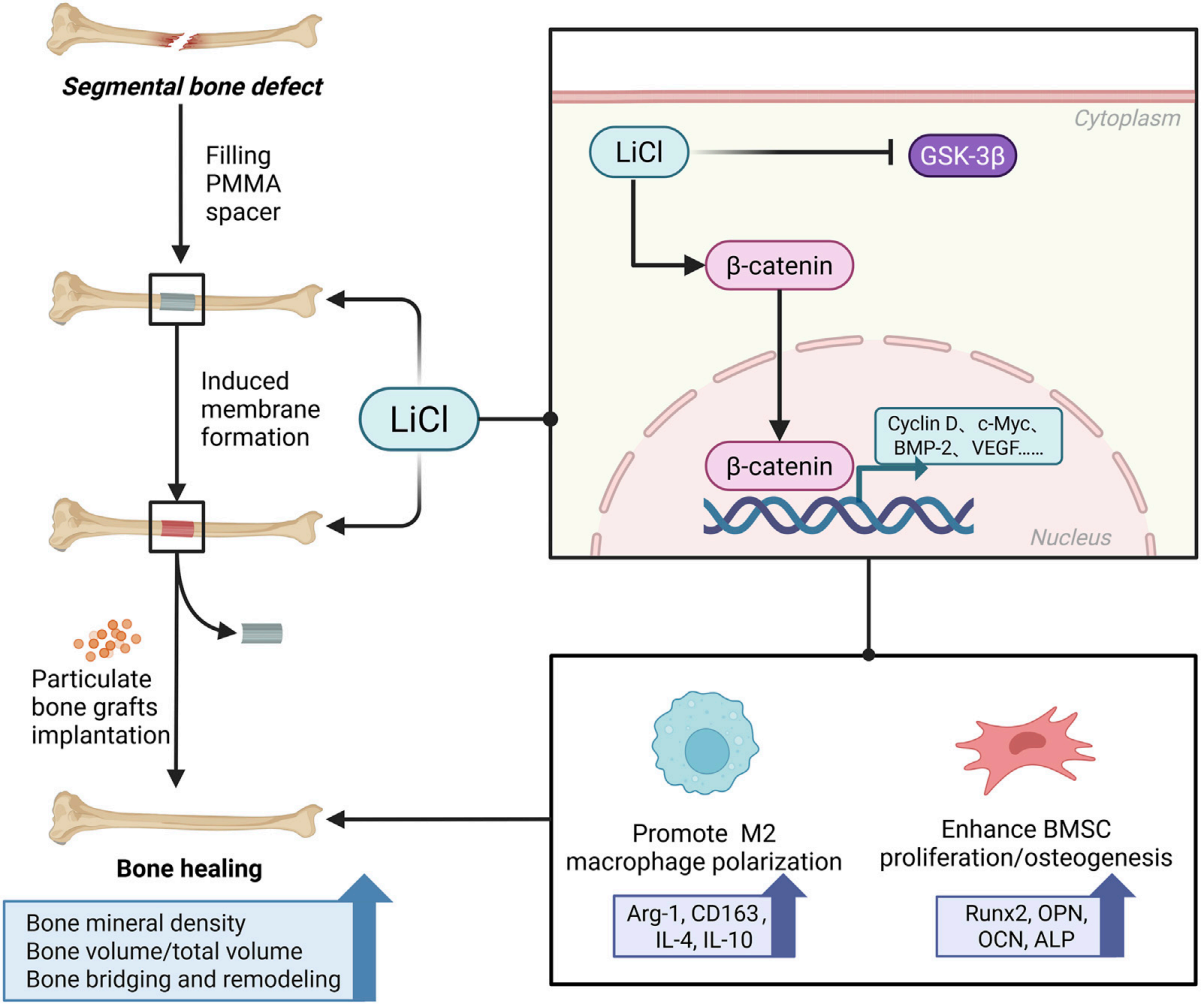
A schematic diagram displaying the underlying mechanism of LiCl promoting bone healing in IMT. We demonstrated that the administration of LiCl may enhance BMSCs differentiation and promote M2 macrophage polarization by activating the Wnt/β-catenin pathway.

The novelty of this study lay in the incorporation of PMMA particles (50–100 nm in diameter) into the culture medium, where they successfully stimulated the microenvironment of BMSCs and macrophages in an *in vivo* IMT. Furthermore, this approach verified the mechanism by which LiCl promoted bone formation in the IMT at the cellular level. Nevertheless, there may have been displacement of PMMA particles in the culture dishes, and the potential impact of mechanical stimulation cannot be ruled out. Qualitative analyses via ALP and ARS staining, and a quantitative Western blot analysis of osteogenic differentiation markers revealed that PMMA particles promoted the osteogenic differentiation of BMSCs prior to administration of LiCl, which is consistent with our *in vivo* IMT observations in rats. However, the underlying mechanisms require further investigation. Subsequent supplementation with LiCl produced a concentration-dependent increase in BMSC osteogenic differentiation in the PMMA particle-induced microenvironments. The activity of Wnt/β-Catenin pathway is constitutively suppressed by the active form of GSK3b under basal condition (Tarchala et al., 2016). The inactivation (phosphorylation) of GSK3b leads to dephosphorylation of β-Catenin and activation of Wnt/β-Catenin pathway to promote bone anabolism (Uccelli et al., 2008). This pathway has been regarded as the most implicated signaling mechanism in the regulation of osteoblastogenesis and bone formation by LiCl due to its well-established anti-GSK3b property (Wang et al., 2023; Wije et al., 2014). Based on the detection of Wnt pathway-related downstream proteins and gene expression in BMSCs, we suggest that LiCl promotes BMSC osteogenesis via Wnt pathway activation (Figure 10).

A key finding of our study was that LiCl significantly promoted the polarization of M2 macrophages and inhibited M1 polarization. This was confirmed by the downregulated expression of CCR7 and CD86 that occurred concomitant with the upregulated expression of Arg-1 and CD163. The effect of LiCl was also associated with elevated levels of anti-inflammatory cytokines (IL-10, IL-4) and decreased levels of pro-inflammatory cytokines (TNF-α, IL-6), further suggesting that LiCl creates a microenvironment more conducive to bone repair. This finding emphasizes the pivotal function of macrophage polarization in determining the quality of the IM, and suggests that LiCl may augment the bioactivity of the IM and provide a more favorable environment for bone graft integration.

Although PMMA induces a pro-inflammatory activation and leads to the M1 polarization of macrophages, as indicated by increased CCR7 and CD86 expression, it positively influences the Wnt/β-catenin pathway, and thereby promotes osteogenic differentiation. This may be due to the fact that PMMA induces a low-concentration, sterile inflammatory response (Wong et al., 2020). Such mild inflammation not only effectively promotes IM formation but also facilitates osteogenesis under appropriate conditions (Yang et al., 2019; Ziroglu et al., 2023). Therefore, despite PMMA being a pro-inflammatory factor, it may, to some extent, enhance bone repair by inducing a low-grade inflammatory response.

Macrophage-BMSC crosstalk might also enhance the therapeutic efficacy of the IMT. We found that the implantation of PMMA spacers initiated foreign body responses, which induced M1 macrophages to secrete inflammatory cytokines and recruit BMSCs to the sites of bone defects. Within the IM, macrophages transitioned to M2 phenotypes with tissue-regenerative capacity, and collaborated with BMSCs, osteoblasts, and osteoclasts to facilitate bone regeneration after grafting. A previous study demonstrated that substituting titanium for spacers in the IMT reduced foreign body responses, and impaired bone regeneration (Hachim et al., 2017). That finding highlighted the critical roles of foreign body responses and macrophage-BMSC crosstalk in promoting bone regeneration via the IMT.

*In vivo* results further corroborated these observations, by showing that LiCl treatment could enhance bone regeneration in a large bone defect model. Micro-CT and histological analyses revealed that high-dose LiCl significantly upregulated the BV/TV ratio and BMD in the high-dose LiCl group when compared to the control group, resulting in a more organized trabecular bone formation. Additionally, X-ray imaging and histological staining confirmed enhanced bone bridging and cortical bone remodeling in the LiCl-treated groups. These findings indicate that LiCl not only enhances the biological attributes of the IMs but also promotes the integration of bone grafts and new bone formation.

Despite these promising results, our study does have some limitations. First, the PMMA particles (50–100 nm in diameter) were co-cultured with RAW264.7 macrophages, but transmission electron microscopy was not employed to examine cellular morphology or phagocytosis. Consequently, it is unclear whether PMMA provided for intracellular or extracellular stimulation of the RAW264.7 macrophages. Second, all *in vivo* assessments were performed at a single time point (12 weeks). Intermediate evaluations (e.g., 2 or 4 weeks) would help depict the dynamic bone healing process under LiCl treatment. Lastly, we only detected the expression of the downstream proteins β-catenin, cyclin D, and c-myc in the Wnt/β-catenin pathway. Incorporation of a specific inhibitor of the Wnt/β-catenin pathway (e.g., using DKK1, XAV939, or siRNA) in both stimulatory and inhibitory experiments may verify the role of LiCl in activating the Wnt/β-catenin pathway in future studies.

While our results suggest that LiCl has beneficial effects at specific concentrations, its potential toxicity and systemic effects need to be further evaluated in future clinical translation studies.

## Conclusion

In conclusion, this study demonstrated that LiCl enhances the biological properties of IMs by regulating macrophage polarization and promoting an osteogenic microenvironment. Furthermore, LiCl accelerates bone defect healing by enhancing BMSC differentiation and new bone regeneration, likely via activation of the Wnt/β-catenin pathway. These findings suggest LiCl as a promising adjunct for improving bone defect repair outcomes. However, further investigations are required to refine the dosing regimens required and assess the long-term safety profile of LiCl in clinical settings.

## Data Availability

The original contributions presented in the study are included in the article/supplementary material, further inquiries can be directed to the corresponding author.
